# Long noncoding RNA associations with an IFI27–MCEMP1 transcript pattern in Kawasaki disease

**DOI:** 10.3389/fimmu.2026.1882860

**Published:** 2026-09-23

**Authors:** Fang Liu, Xing Xue, Zhi Han, Bo Jin, Weiwei Li, Naoto Ozawa, Takumi Ichikawa, Ellen Ling, Xinyang Zhao, Henry Chubb, Scott R. Ceresnak, Gary L. Darmstadt, Doff B. McElhinney, Harvey J. Cohen, Seda Tierney, Xuefeng B. Ling

**Affiliations:** 1 Heart Center, Children’s Hospital of Fudan University, Shanghai, China; 2 School of Medicine, Stanford University, Stanford, CA, United States; 3 OncoOmicsDx Clinical Laboratory, Rockville, MD, United States; 4 Nippon Life Insurance Company, Osaka, Japan; 5 Florida State University, Tallahassee, FL, United States; 6 University of Kansas Medical Center, Kansas City, KS, United States

**Keywords:** CHROMR, IFI27, Kawasaki disease, long non coding RNA (lncRNA), MALAT1, MCEMP1, peripheral blood mononuclear cells, transcriptomic biomarkers

## Abstract

Kawasaki disease (KD) is an acute pediatric vasculitis with risk of coronary artery injury. A two-transcript IFI27–MCEMP1 score has distinguished KD from other febrile illnesses, but its relationship to long noncoding RNAs (lncRNAs) is incompletely defined. We evaluated whether peripheral blood mononuclear cell (PBMC) lncRNA expression differed between KD and febrile controls and was associated with the IFI27–MCEMP1 transcript pattern. Children younger than 8 years were prospectively enrolled at the Children’s Hospital of Fudan University during 2024–2025. The newly enrolled cohort included 55 children with KD and 48 febrile controls; integration with two same-site cohorts yielded 188 children with KD and 175 febrile controls. Bulk-PBMC expression of IFI27, MCEMP1, CHROMR, MALAT1, and NEAT1 was measured by reverse transcription quantitative PCR and normalized to GAPDH. The prespecified score distinguished KD from febrile controls with areas under the receiver operating characteristic curve of 0.88 (95% CI, 0.80–0.95) in the newly enrolled cohort and 0.89 (95% CI, 0.86–0.93) in the integrated cohort. In unadjusted integrated analyses, CHROMR and MALAT1 expression was lower in KD, whereas NEAT1 did not differ significantly. CHROMR ΔCt correlated with IFI27 ΔCt (r=0.55; Holm-adjusted P<.001). After adjustment for fever duration and a CBC-derived monocyte fraction, lower CHROMR and IFI27 and higher MCEMP1 expression remained significant, whereas the MALAT1 difference was attenuated. Exact fever-day matching yielded concordant CHROMR, IFI27, and MCEMP1 group differences, but the adjusted CHROMR–IFI27 correlation was not significant in the restricted matched population. Thus, KD was associated with a bulk-PBMC transcript pattern involving CHROMR, IFI27, and MCEMP1; the MALAT1 finding was less robust. These associations motivate hypotheses about interferon-associated and myeloid-associated biology but do not establish pathway activity or causality. Multicenter studies with standardized sampling, cell-resolved profiling, and independent CHROMR assay confirmation are required.

## Introduction

Kawasaki disease (KD) is an acute systemic vasculitis of childhood and a leading cause of acquired heart disease among children in developed countries (1, 2). Timely treatment with intravenous immunoglobulin substantially reduces the risk of coronary artery aneurysms (3). KD is characterized by prolonged fever, mucocutaneous inflammation, systemic inflammatory findings, and a predilection for medium-sized arteries, particularly the coronary arteries (4). Although the clinical phenotype and vascular complications are well recognized, the cause and immunopathogenesis of KD remain incompletely understood. The acute illness is accompanied by changes in innate and adaptive immune responses, inflammatory mediators, endothelial biology, and vascular remodeling (1, 2, 4). However, the circulating transcriptional programs that distinguish KD from other pediatric febrile illnesses remain only partly defined.

A central clinical and biological challenge is the substantial overlap between KD and viral, bacterial, or other inflammatory febrile illnesses. Children with incomplete or evolving KD may not initially fulfill the complete clinical criteria, whereas children with other febrile illnesses may present with overlapping mucocutaneous findings and elevated inflammatory markers (4, 5). This overlap complicates diagnosis but also provides a stringent setting in which to investigate disease-associated host-response patterns. Blood transcript measurements may identify molecular differences not apparent from clinical findings or conventional inflammatory markers alone and may help connect diagnostic signals with broader immune-transcriptional states.

We previously validated a two-gene whole-blood reverse transcription quantitative polymerase chain reaction assay measuring IFI27 and MCEMP1 for distinguishing KD from other febrile illnesses (6). IFI27 encodes an interferon-stimulated gene that can counter-regulate antiviral innate immune responses by interfering with RIG-I signaling (7). MCEMP1 has been associated with inflammatory myeloid states and is expressed in CD14-positive cells, classical monocytes, and macrophages; experimental studies outside KD have implicated it in cellular chemotaxis, adhesion, and migration (8, 9). Nevertheless, IFI27 abundance alone is not a quantitative measure of global interferon signaling, and MCEMP1 abundance alone does not demonstrate myeloid-cell activation or distinguish altered transcription within cells from differences in cell abundance. Accordingly, we use the term “IFI27–MCEMP1 transcript pattern” to describe the observed combination of lower IFI27 and higher MCEMP1 expression in KD relative to febrile controls. Any broader interferon–myeloid interpretation of this pattern is conceptual and hypothesis-generating rather than evidence of a demonstrated biological pathway.

Long noncoding RNAs (lncRNAs) can influence immune-responsive gene expression through diverse and transcript-specific mechanisms, including transcriptional regulation, chromatin organization, protein sequestration, and modulation of inflammatory signaling. CHROMR is a primate-specific lncRNA induced by viral infection. In human macrophages, CHROMR supports interferon-stimulated gene expression by sequestering the transcriptional corepressor IRF2BP2 and thereby licensing IRF-dependent transcription (10). NEAT1 is an architectural component of nuclear paraspeckles and can promote immune-responsive IL8 expression through stimulus-dependent relocation of the transcriptional repressor SFPQ from the IL8 promoter to paraspeckles (11). MALAT1 can interact with nuclear NF-κB and modulate lipopolysaccharide-induced inflammatory cytokine expression in macrophages (12). These findings identify biologically plausible links between selected lncRNAs and immune-responsive transcription, but they were established in experimental settings outside KD and do not demonstrate that the same mechanisms operate in KD.

CHROMR, MALAT1, and NEAT1 therefore provided a focused candidate panel for evaluating whether selected lncRNA changes accompany the IFI27–MCEMP1 transcript pattern in KD. Importantly, measurements from bulk peripheral blood mononuclear cells (PBMCs) may reflect both transcript regulation within individual cell types and differences in PBMC composition. Transcript abundance may also vary with fever duration and inflammatory stage. Associations among these transcripts must therefore be interpreted as exploratory molecular relationships rather than direct regulatory, cell-intrinsic, or causal effects.

We hypothesized that selected PBMC-derived lncRNAs would differ between children with KD and febrile controls and would be associated with IFI27 or MCEMP1 transcript abundance. To test this hypothesis, we profiled CHROMR, MALAT1, and NEAT1 together with IFI27 and MCEMP1 in a newly enrolled cohort and integrated these data with two previously characterized same-site cohorts. We evaluated the reproducibility of the prespecified IFI27–MCEMP1 score, differential expression of the candidate lncRNAs, and associations between the lncRNAs and each component of the two-transcript pattern. The study was designed to characterize an observed bulk-PBMC RNA pattern and generate testable hypotheses for temporally standardized, cell-resolved, and functional studies, rather than to establish altered interferon signaling, functional myeloid activation, direct lncRNA regulation, or a causal pathway to coronary artery injury.

## Methods

### Study design and analytic objectives

We conducted a diagnostic-validation and exploratory biomarker-association study in children evaluated for suspected Kawasaki disease (KD). We assessed reproducibility of the previously validated IFI27–MCEMP1 reverse transcription quantitative polymerase chain reaction (RT-qPCR) score (6) in a newly enrolled KD and febrile-control cohort and evaluated whether selected peripheral blood mononuclear cell (PBMC) long noncoding RNAs (lncRNAs) differed between groups and were associated with the observed transcript pattern. Additional exploratory sensitivity analyses examined PBMC composition, fever duration, and CHROMR amplification efficiency. The study characterized transcript associations; it did not test pathway activity, cell-intrinsic regulation, causal mediation, or incremental diagnostic utility.

### Study population and PBMC transcriptomic cohorts

Children younger than 8 years were prospectively enrolled at the Children’s Hospital of Fudan University, Shanghai, China, between 2024 and 2025. The newly enrolled validation cohort (batch 3) included 55 children with KD and 48 febrile controls. For integrated lncRNA and diagnostic-score analyses, batch 3 was combined with two previously characterized same-site cohorts generated using comparable enrollment criteria, sample-processing procedures, and RT-qPCR protocols. Batch 1 included 53 children with KD and 81 febrile controls, and batch 2 included 80 children with KD and 46 febrile controls. The integrated same-site cohort therefore comprised 188 children with KD and 175 febrile controls (363 participants). Cohort assembly and the analytic use of each cohort are summarized in **Table 1** and **Figure 1**.

**Table 1:** Cohort assembly and analytic samples.

Cohort component	Source/role in present study	KD, No.	Febrile controls, No.	Total, No.	Primary use
Batch 1	Previously characterized same-site cohort	53	81	134	Integrated lncRNA and diagnostic-score analyses
Batch 2	Previously characterized same-site cohort	80	46	126	Integrated lncRNA and diagnostic-score analyses
Batch 3	Newly enrolled validation cohort	55	48	103	Independent diagnostic validation and lncRNA profiling
Integrated same-site cohort	Batches 1–3 combined	188	175	363	Primary integrated lncRNA, correlation, and diagnostic-score analyses

FC, febrile control; KD, Kawasaki disease; lncRNA, long noncoding RNA; PBMC, peripheral blood mononuclear cell; RT-qPCR, reverse transcription quantitative polymerase chain reaction.

Counts represent unique children. Batch 3 was the newly enrolled same-site validation cohort; batches 1 and 2 were previously characterized same-site cohorts. The integrated cohort combined batches 1–3 for application of the prespecified diagnostic score and for exploratory candidate-lncRNA analyses. The score and diagnostic threshold were not refitted. PBMC RNA was profiled by RT-qPCR for IFI27, MCEMP1, CHROMR, MALAT1, NEAT1, and GAPDH.

**Figure 1:**
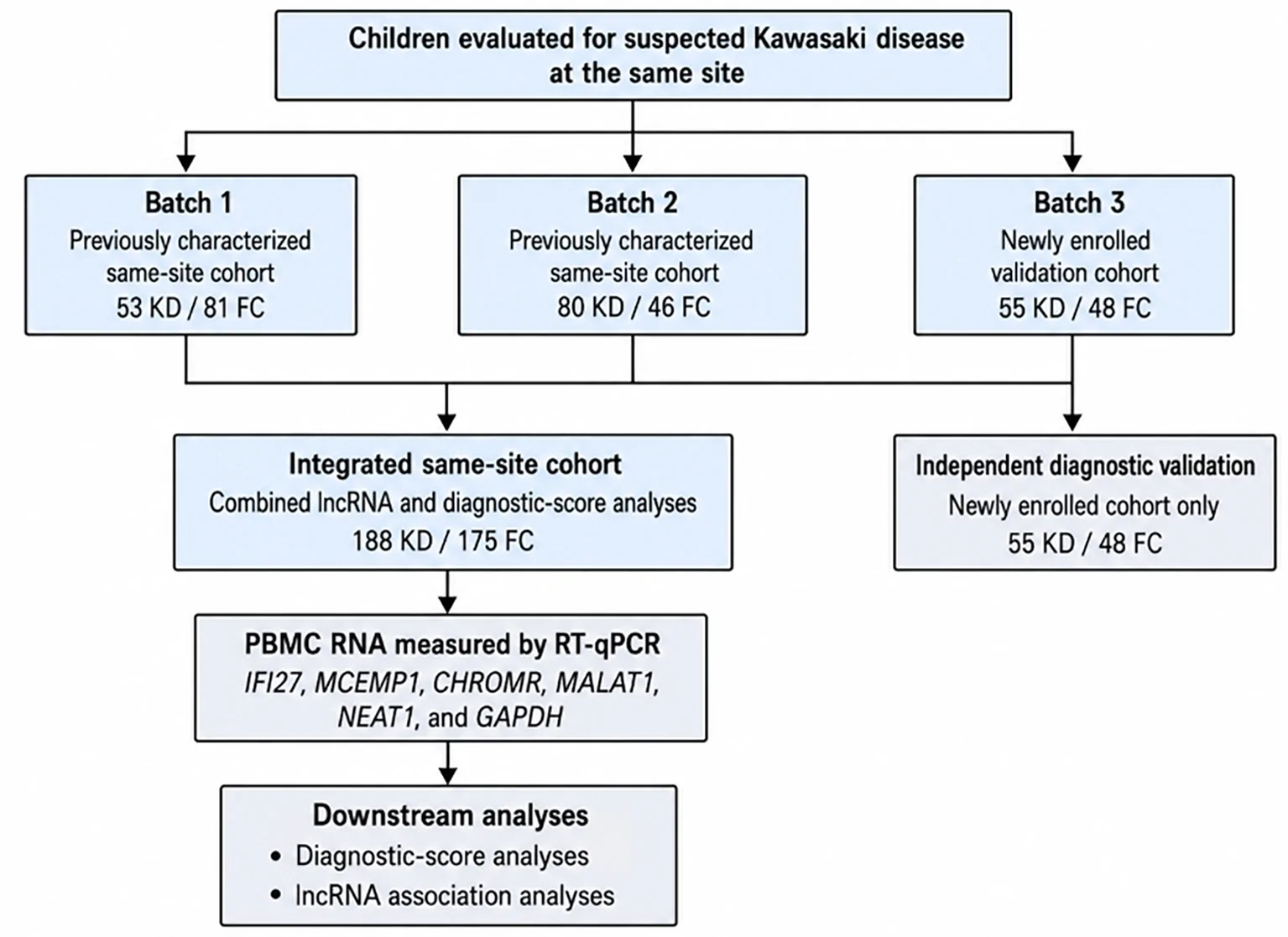
Study design and PBMC RNA profiling cohorts. Flow diagram showing assembly and analytic use of the newly enrolled validation cohort and the integrated same-site cohort. Batch 3 comprised 55 children with Kawasaki disease (KD) and 48 febrile controls (FC) and was evaluated separately for newly enrolled same-site diagnostic validation. Batches 1 and 2 were previously characterized same-site cohorts comprising 53 KD/81 FC and 80 KD/46 FC participants, respectively. Combining all three batches yielded 188 KD and 175 FC participants for application of the prespecified diagnostic score and exploratory candidate-lncRNA analyses. PBMC RNA was measured by RT-qPCR for IFI27, MCEMP1, CHROMR, MALAT1, NEAT1, and GAPDH. Counts represent unique children. FC, febrile control; IFI27, interferon alpha-inducible protein 27; KD, Kawasaki disease; lncRNA, long noncoding RNA; MCEMP1, mast cell-expressed membrane protein 1; PBMC, peripheral blood mononuclear cell; RT-qPCR, reverse transcription quantitative polymerase chain reaction.

### Clinical classification and variables

Eligible participants were children presenting with fever and clinical concern for KD or children evaluated for alternative febrile illnesses. KD was diagnosed according to the 2017 American Heart Association criteria for complete or incomplete KD (4). Classification was based on clinical features, laboratory findings, and echocardiographic evaluation. Complete KD was defined by fever with sufficient principal clinical criteria. Incomplete KD was diagnosed when complete clinical criteria were not fulfilled but guideline-supported clinical, laboratory, or echocardiographic criteria were met.

Febrile controls were children evaluated for alternative febrile illnesses who did not meet diagnostic criteria for KD. Control diagnoses were grouped as viral, bacterial, or other inflammatory febrile illness on the basis of clinical assessment, laboratory testing, microbiologic studies when available, and treating-physician adjudication. Some historical febrile-control subgroup annotations were non-mutually exclusive; comparator-specific analyses in the integrated cohort were therefore interpreted as subgroup contrasts rather than prevalence estimates. Children were excluded if clinical information was insufficient for diagnostic classification or RNA quantity or quality was inadequate for RT-qPCR analysis.

Demographic, clinical, and laboratory variables were abstracted from the clinical record. These included age, sex, fever duration at sample collection, hemoglobin concentration, C-reactive protein concentration, platelet count, white blood cell count, leukocyte differential percentages, incomplete-KD status, coronary artery status, and febrile-control diagnostic subgroup. Clinical and laboratory characteristics of the newly enrolled cohort are reported in **Table 2**.

**Table 2:** Demographic and clinical features of the newly enrolled cohort.

Characteristic	KD (n = 55)	Febrile controls (n = 48)	P value
Age, y, median (IQR)	2.25 (1.25–3.42)	3.79 (1.98–5.35)	<.001
Sex, No. (%)			.793
Female	17 (30.9)	16 (33.3)	
Male	38 (69.1)	32 (66.7)	
Fever duration at sample collection, d, median (IQR)	6 (5–6)	2 (1–3)	<.001
Hemoglobin, g/L, mean (SD)	109.9 (10.2)	119.4 (10.2)	<.001
C-reactive protein, mg/L, median (IQR)	71.5 (48.6–102.7)	13.2 (7.8–38.7)	<.001
Platelet count, 10³/μL, median (IQR)	243.0 (239.0–249.0)	237.5 (197.5–292.8)	.070
White blood cell count, 10³/μL, median (IQR)	17.4 (12.2–19.6)	8.6 (7.1–13.8)	<.001
Incomplete KD, No. (%)	21 (38.2)	—	—
Coronary artery status, No. (%)		—	—
Normal	36 (65.5)	—	—
Dilated or aneurysmal	19 (34.5)	—	—
Febrile-control diagnostic subgroup, No. (%)	—		—
Viral febrile illness	—	15 (31.3)	—
Bacterial febrile illness	—	20 (41.7)	—
Other inflammatory illness	—	13 (27.1)	—

FC, febrile control; IQR, interquartile range; KD, Kawasaki disease.

Values are presented as indicated in the row labels. Age was converted from months to years. All P values are two-sided and unadjusted; t tests were used for approximately normally distributed continuous variables, Wilcoxon rank-sum tests for skewed continuous variables, and chi-square or Fisher exact tests for categorical variables, as appropriate. Febrile-control diagnostic subgroups were mutually exclusive in the newly enrolled cohort and use n=48 as the denominator. Coronary artery status is reported only for participants with KD.

### Ethics approval and consent

The study was approved by the institutional review board of the Children’s Hospital of Fudan University. Written informed consent was obtained from the parent or legal guardian of each participant before study procedures. The study was conducted in accordance with applicable ethical standards for human-participant research.

### Blood processing, RNA extraction, and RT-qPCR

Peripheral blood samples were obtained during the acute febrile illness and, for children diagnosed with KD, before intravenous immunoglobulin treatment. PBMCs were isolated using standardized laboratory procedures and processed for total RNA extraction. RNA quantity and purity were assessed spectrophotometrically, and RNA quality was verified before reverse transcription. Complementary DNA was synthesized from total RNA using random-hexamer priming.

Expression of IFI27, MCEMP1, CHROMR, MALAT1, NEAT1, and GAPDH was measured by RT-qPCR. Reactions were performed in technical duplicate using SYBR Green chemistry on a real-time PCR platform. GAPDH served as the endogenous reference transcript. No-template controls were included to monitor contamination, and post-amplification melt-curve analysis was used to assess whether each assay produced a single predominant product. Reactions included in the analysis showed a single dominant assay-specific melting peak. Representative MALAT1 and NEAT1 melt curves are provided in **Supplementary Figure S1**, and a representative archived CHROMR melt curve is provided in **Supplementary Figure S4**. IFI27 and MCEMP1 assay specifications were reported previously (6). Primer sequences, amplicon sizes, slopes, amplification efficiencies, and coefficients of determination for GAPDH, CHROMR, NEAT1, and MALAT1 are provided in **Table 3**.

**Table 3:** RT-qPCR primer sequences and assay-performance characteristics for lncRNA profiling.

Gene	Forward primer, 5′–3′	Reverse primer, 5′–3′	Amplicon size, bp	Slope	Efficiency, %	R²
GAPDH	GAAGGTGAAGGTCGGAGTC	GAAGATGGTGATGGGATTTC	226	−3.474	94.041	0.9940
CHROMR	GCAGGAGCTTGAATTTCAGT	TGTACTGAGTGGGCATTTAT	91	−3.406	96.626	0.9982
NEAT1	AGAGCATGGTAACCACAGAT	TATGGCAAAGTACTCCCCAC	169	−3.303	100.796	0.9980
MALAT1	GGTTCAGAAGGTCTGAAGCTC	CCCAGAAGTGTTTACACTGCT	103	−3.287	101.479	0.9470

Ct, cycle threshold; lncRNA, long noncoding RNA; qPCR, quantitative polymerase chain reaction; R², coefficient of determination; RT-qPCR, reverse transcription quantitative polymerase chain reaction.

Amplification efficiency was calculated as E (%) = [10^(−1/slope) − 1] × 100 from the regression of Ct on log10-transformed relative cDNA input. Six sequential threefold cDNA dilutions were measured in technical duplicate. For CHROMR, regression was restricted to the contiguous linear range (S2–S4) using unrounded mean Ct values, yielding a slope of −3.406, R²=0.9982, and an amplification efficiency of 96.626% (approximately 96.6%); technical-replicate SDs were 0.142–0.391 Ct. The remaining dilution levels are shown in **Supplementary Figure S4**. The primary analyses used target-minus-GAPDH ΔCt values without a separate gene-specific efficiency correction; the assay-efficiency-corrected sensitivity analysis did not materially change the CHROMR findings. The archived CHROMR melt curve showed a single predominant peak, but amplicon identity was not confirmed by an archived gel or sequencing record.

Amplification efficiency was calculated from the slope of the regression of cycle threshold (Ct) against log10-transformed relative template input using E (%) = [10^(−1/slope) − 1] × 100. The archived CHROMR standard curve consisted of six sequential threefold cDNA dilutions measured in technical duplicate. The contiguous dilution levels within the linear quantitative range were retained for regression; the remaining levels are displayed in **Supplementary Figure S4** for transparency. Technical-replicate SDs across the retained range were 0.142 to 0.391 Ct. The re-estimated CHROMR efficiency was approximately 96.6%, compared with 94.0% for GAPDH. The primary analyses used target-minus-GAPDH ΔCt values without a separate gene-specific efficiency correction. An additional sensitivity analysis calculated CHROMR abundance using assay-specific amplification factors derived from the efficiencies for CHROMR and GAPDH. Because no archived agarose-gel image or Sanger-sequencing record was available, the CHROMR melt curve supports amplification of a predominant product but does not provide sequence-level confirmation of amplicon identity.

### Transcript normalization and IFI27–MCEMP1 score

For the principal analyses, transcript abundance was normalized to GAPDH as ΔCt = Ct(target) − Ct(GAPDH). Higher ΔCt values indicate lower relative expression. The prespecified KD score was calculated as KD score = ΔCt(IFI27) − ΔCt(MCEMP1). Higher scores represent the observed KD-associated transcript pattern of relatively lower IFI27 and higher MCEMP1 expression. The diagnostic-positive operating point of KD score ≥2.5 was carried forward from the prior validation study (6) and applied without recalibration in both the newly enrolled and integrated same-site cohorts. No model refitting, coefficient adjustment, or *post hoc* threshold optimization was performed. Diagnostic performance is reported in **Table 4** and **Figure 2**.

**Table 4:** Reproducibility of the prespecified IFI27–MCEMP1 score across febrile-control comparators.

Cohort	Comparator	n	AUC (95% CI)	Sensitivity, % (95% CI)	Specificity, % (95% CI)	PPV, % (95% CI)	NPV, % (95% CI)
Newly enrolled cohort	All FC	55 KD/48 FC	0.88 (0.80–0.95)	81.8 (69.1–90.9)	87.5 (74.8–95.3)	88.2 (76.1–95.6)	80.8 (67.5–90.4)
Integrated same-site cohort	All FC	188 KD/175 FC	0.89 (0.86–0.93)	85.6 (79.8–90.3)	84.6 (78.4–89.6)	85.6 (79.8–90.3)	84.6 (78.4–89.6)
Newly enrolled cohort	Viral FC	55 KD/15 FC	0.97 (0.93–1.00)	81.8 (69.1–90.9)	100.0 (78.2–100.0)	—	—
Newly enrolled cohort	Bacterial FC	55 KD/20 FC	0.79 (0.65–0.93)	81.8 (69.1–90.9)	75.0 (50.9–91.3)	—	—
Newly enrolled cohort	Other inflammatory FC	55 KD/13 FC	0.90 (0.79–1.00)	81.8 (69.1–90.9)	92.3 (64.0–99.8)	—	—
Integrated same-site cohort	Viral FC	188 KD/62 FC	0.96 (0.93–0.98)	85.6 (79.8–90.3)	95.2 (86.5–99.0)	—	—
Integrated same-site cohort	Bacterial FC	188 KD/60 FC	0.81 (0.73–0.88)	85.6 (79.8–90.3)	75.0 (62.1–85.3)	—	—
Integrated same-site cohort	Other inflammatory FC	188 KD/55 FC	0.92 (0.87–0.96)	85.6 (79.8–90.3)	83.6 (71.2–92.2)	—	—

AUC, area under the receiver operating characteristic curve; CI, confidence interval; FC, febrile control; KD, Kawasaki disease; NPV, negative predictive value; PPV, positive predictive value.

KD score = ΔCt(IFI27) − ΔCt(MCEMP1). The prespecified diagnostic-positive operating point was KD score ≥2.5; the score and threshold were applied without refitting or recalibration. Sensitivity is repeated across comparator-specific rows because the same KD cases were compared with each FC subgroup within each cohort. PPV and NPV are reported only for the full FC comparison because predictive values depend on the case-control composition and may not generalize to clinical prevalence. AUC 95% CIs were estimated by the DeLong method; CIs for sensitivity, specificity, PPV, and NPV used exact binomial methods. Two historical integrated-cohort FC labels were non-mutually exclusive; comparator-specific analyses are therefore subgroup contrasts rather than prevalence categories, and subgroup counts may exceed the number of unique FC participants.

**Figure 2:**
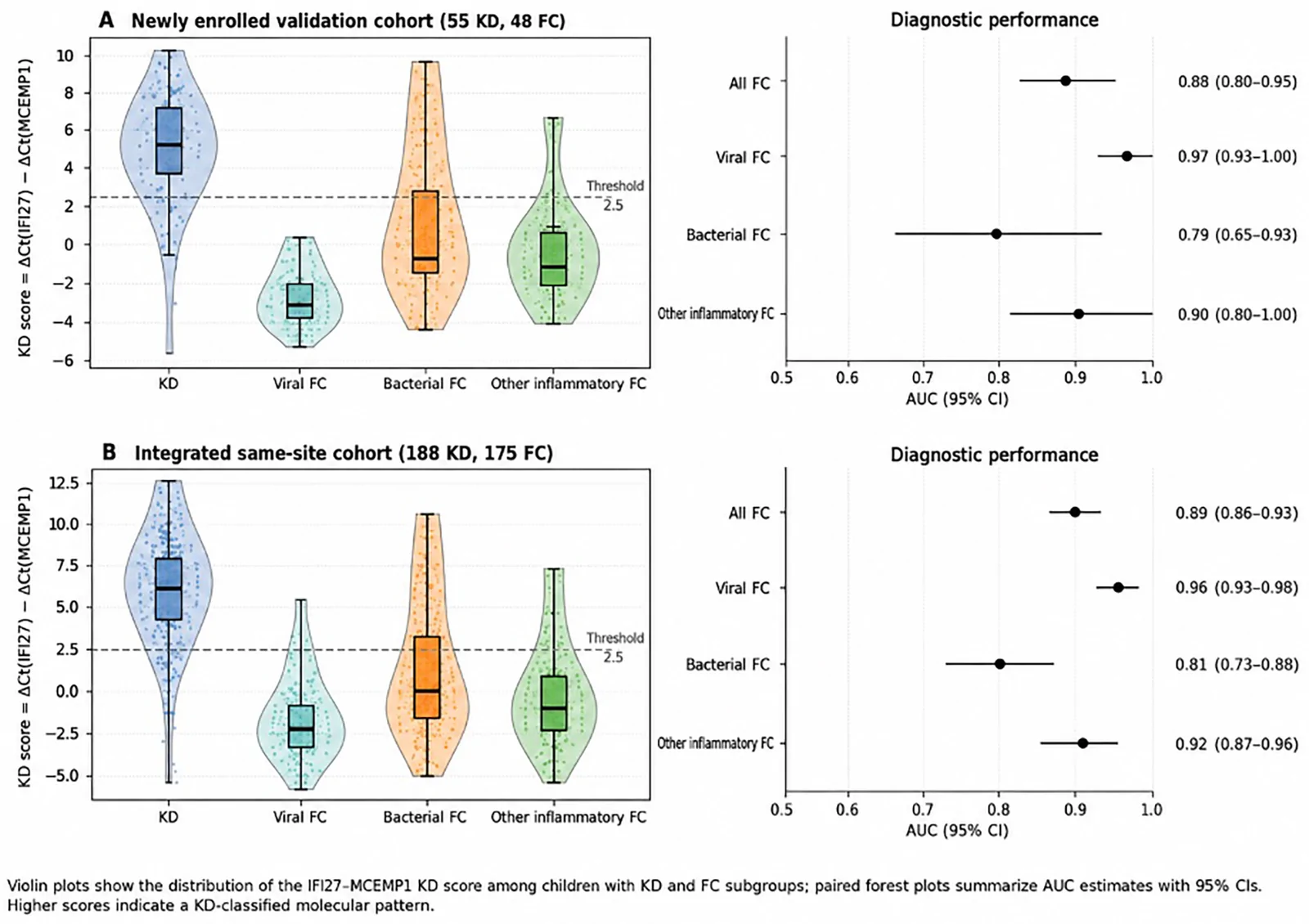
Diagnostic performance of the prespecified IFI27–MCEMP1 score. Violin plots with embedded boxplots show the IFI27–MCEMP1 KD-score distributions, and forest plots show AUCs with 95% CIs. The dashed horizontal line marks the prespecified diagnostic-positive threshold of 2.5. **(A)** Newly enrolled validation cohort (55 KD and 48 FC participants): AUC, 0.88 (95% CI, 0.80–0.95) for all FC; 0.97 (0.93–1.00) for viral FC; 0.79 (0.65–0.93) for bacterial FC; and 0.90 (0.79–1.00) for other inflammatory FC. **(B)** Integrated same-site cohort (188 KD and 175 FC participants): AUC, 0.89 (95% CI, 0.86–0.93) for all FC; 0.96 (0.93–0.98) for viral FC; 0.81 (0.73–0.88) for bacterial FC; and 0.92 (0.87–0.96) for other inflammatory FC. The score and threshold were applied without refitting or recalibration. Higher scores indicate the observed KD-associated transcript pattern of relatively lower IFI27 and higher MCEMP1 expression. AUC, area under the receiver operating characteristic curve; CI, confidence interval; FC, febrile control; KD, Kawasaki disease.

### Candidate lncRNA selection and outcomes

CHROMR, MALAT1, and NEAT1 were selected before analysis based on evidence from non-KD systems. CHROMR supports interferon-stimulated gene transcription in human macrophages through an IRF2BP2/IRF-dependent mechanism (10); NEAT1 contributes to paraspeckle organization and stimulus-responsive IL8 transcription (11); and MALAT1 interacts with NF-κB and modulates lipopolysaccharide-induced inflammatory responses (12). These sources motivated candidate selection but were not assumed to establish lncRNA function in KD.

Primary biomarker-association outcomes were KD-versus-febrile-control differences in CHROMR, MALAT1, and NEAT1 and pairwise associations of each lncRNA with IFI27 or MCEMP1. Additional outcomes included candidate-lncRNA and clinical-variable associations with the composite KD score. The secondary diagnostic outcome was performance of the prespecified IFI27–MCEMP1 score in the newly enrolled cohort; integrated-cohort performance assessed same-site reproducibility. Results are presented in **Figures 2**–**5**, **Tables 4**, **5**. **Figure 6** separates measured findings from downstream hypotheses that were not tested.

**Figure 3:**
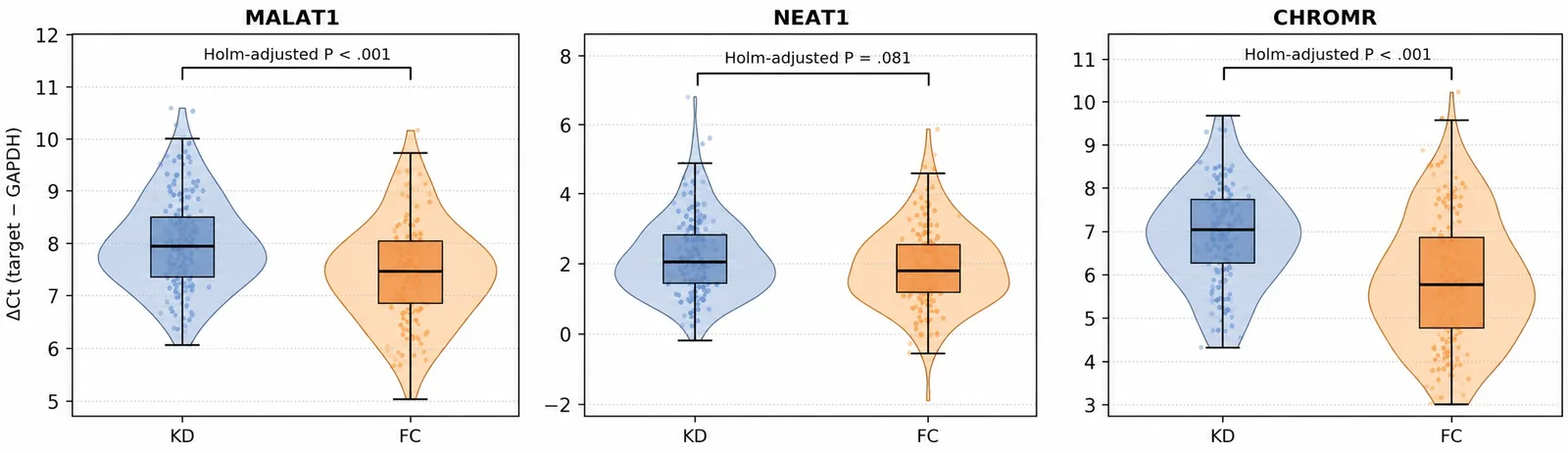
Unadjusted bulk-PBMC candidate-lncRNA differences in Kawasaki disease and febrile controls. Violin plots with embedded boxplots show ΔCt distributions in the integrated same-site cohort of 188 KD and 175 FC participants. A, MALAT1. B, NEAT1. C, CHROMR. Higher ΔCt indicates lower relative expression. Unadjusted group comparisons used two-sided Mann–Whitney U tests with Holm adjustment across the three candidate lncRNAs. MALAT1 and CHROMR ΔCt values were higher in KD than in FC (Holm-adjusted P<.001 for both), whereas NEAT1 did not differ significantly (Holm-adjusted P = .081). In analyses simultaneously adjusted for fever duration and broad PBMC composition, the CHROMR difference remained significant and the MALAT1 estimate was attenuated; complete sensitivity results are reported in **Supplementary Figure S2** and **Supplementary Table S1**. FC, febrile control; KD, Kawasaki disease; lncRNA, long noncoding RNA; PBMC, peripheral blood mononuclear cell.

**Figure 4:**
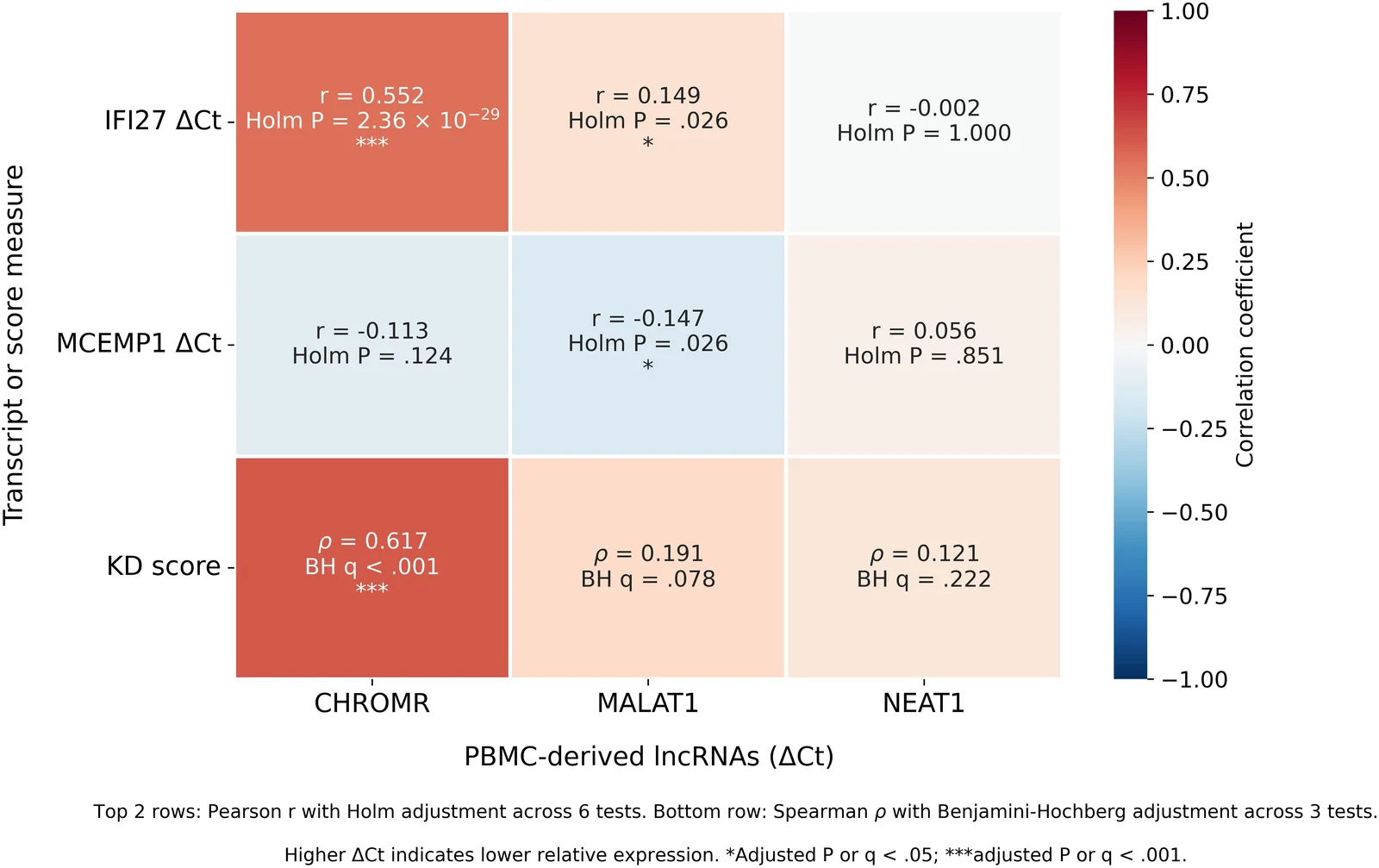
Associations of PBMC-derived lncRNAs with IFI27, MCEMP1, and the prespecified KD score. The top two rows show Pearson correlations between IFI27 or MCEMP1 ΔCt and CHROMR, MALAT1, or NEAT1 ΔCt in the integrated same-site cohort (n=363), with Holm adjustment across six tests. The bottom row shows Spearman correlations between the prespecified KD score and lncRNA ΔCt in the complete paired subset (n=104; 53 KD and 51 FC), with Benjamini–Hochberg adjustment across three tests. Higher ΔCt indicates lower relative expression; therefore, a positive KD-score correlation with lncRNA ΔCt indicates an inverse association between relative lncRNA expression and the KD score. The KD score was associated with CHROMR ΔCt (ρ=0.617; P = 3.19×10^-^¹²; q<.001) but not with MALAT1 ΔCt (ρ=0.191; P = .052; q=.078) or NEAT1 ΔCt (ρ=0.121; P = .222; q=.222). These are unadjusted transcript associations; fever-duration- and PBMC-composition-adjusted CHROMR–IFI27 analyses are reported in **Supplementary Table S1**. FC, febrile control; IFI27, interferon alpha-inducible protein 27; KD, Kawasaki disease; lncRNA, long noncoding RNA; MCEMP1, mast cell-expressed membrane protein 1; PBMC, peripheral blood mononuclear cell.

**Figure 5:**
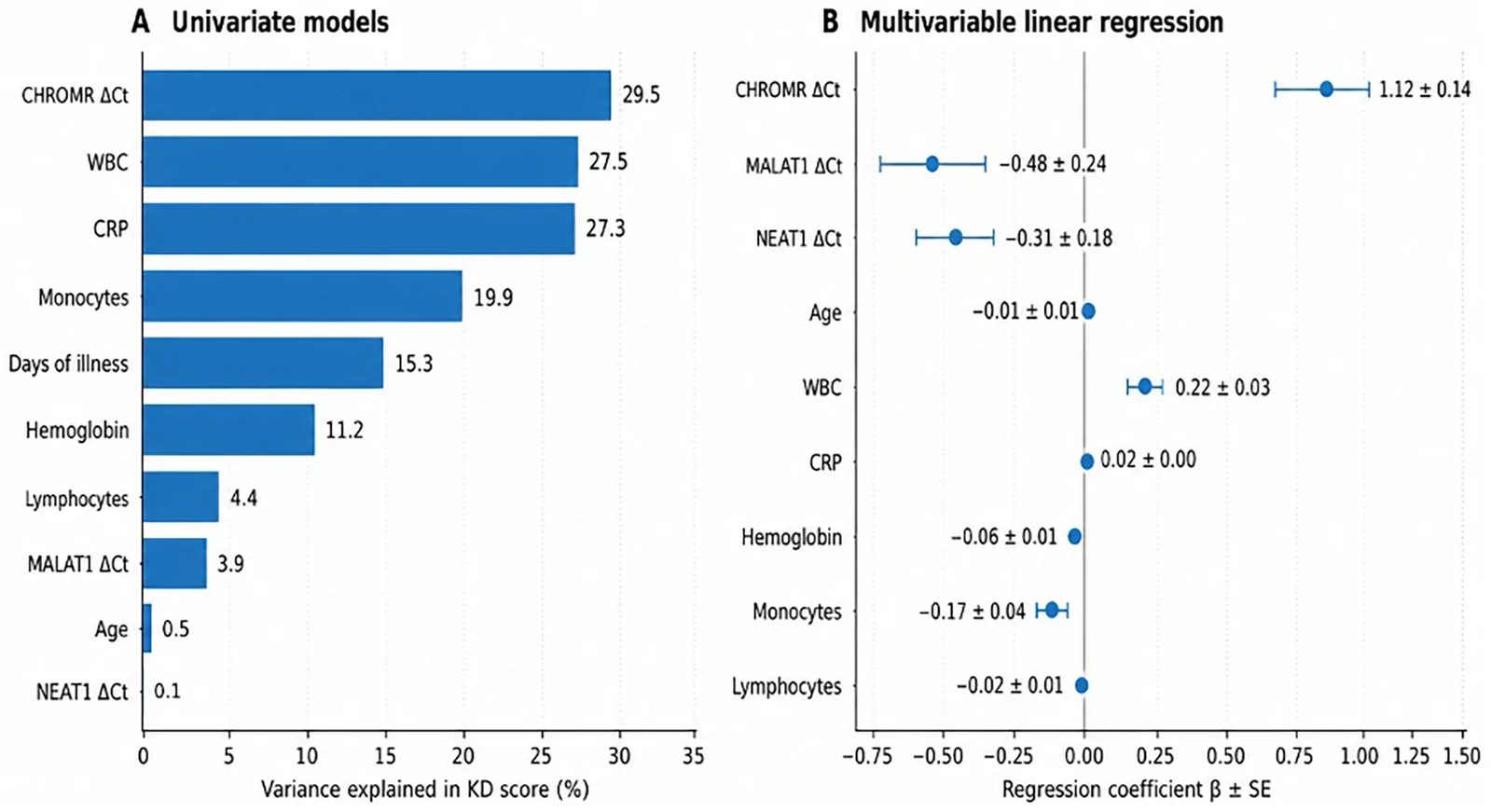
Clinical and lncRNA correlates of the prespecified IFI27–MCEMP1 KD score **(A)** Proportion of KD-score variance (R² × 100) explained by each candidate lncRNA or clinical variable in a separate univariate linear model. **(B)** Multivariable linear-regression coefficients (β) with standard errors for candidate lncRNAs and clinical covariates. Positive coefficients indicate a higher KD score with increasing predictor values, and negative coefficients indicate a lower score. Because higher lncRNA ΔCt denotes lower relative expression, the positive CHROMR ΔCt coefficient corresponds to lower CHROMR expression at higher KD scores. These exploratory models assess association with the existing score; they do not establish incremental diagnostic utility, direct transcriptional regulation, mediation, or causality. FC, febrile control; IFI27, interferon alpha-inducible protein 27; KD, Kawasaki disease; lncRNA, long noncoding RNA; MCEMP1, mast cell-expressed membrane protein 1.

**Table 5:** Summary of candidate-lncRNA group differences, transcript associations, and robustness analyses..

Transcript	KD vs FC expression pattern	Association with IFI27 ΔCt	Association with MCEMP1 ΔCt	Result and interpretive boundary
CHROMR	Higher ΔCt in KD, indicating lower relative expression; Holm-adjusted P <.001	r=0.552; Holm-adjusted P = 2.36×10^-^²^9^	r=−0.113; Holm-adjusted P = .124	Strongest unadjusted lncRNA association was with IFI27. The KD-versus-control CHROMR difference persisted in composition- and fever-adjusted analyses; direct regulation was not tested.
MALAT1	Higher ΔCt in KD, indicating lower relative expression; Holm-adjusted P <.001	r=0.149; Holm-adjusted P = .026	r=−0.147; Holm-adjusted P = .026	Modest unadjusted associations; the KD-versus-control estimate was attenuated after simultaneous adjustment for fever duration and broad PBMC composition (q=.077).
NEAT1	No significant differential-expression signal; Holm-adjusted P = .081	Not significant after multiple-comparison adjustment	Not significant after multiple-comparison adjustment	No significant KD-versus-control difference or pairwise transcript association was detected after multiplicity adjustment.

ΔCt, target-minus-GAPDH cycle-threshold difference; FC, febrile control; IFI27, interferon alpha-inducible protein 27; KD, Kawasaki disease; lncRNA, long noncoding RNA; MCEMP1, mast cell-expressed membrane protein 1; PBMC, peripheral blood mononuclear cell.

Higher ΔCt indicates lower relative expression. Unadjusted group comparisons and IFI27/MCEMP1 correlations used the integrated same-site cohort of 188 KD and 175 FC participants. Group comparisons used two-sided Mann–Whitney U tests with Holm adjustment across three candidate lncRNAs; pairwise transcript associations used Pearson correlations with Holm adjustment across six lncRNA–mRNA tests. Adjusted sensitivity analyses used the quality-controlled matched-data cohort and addressed broad PBMC composition and fever duration. Those analyses supported the CHROMR group difference but attenuated the MALAT1 difference and the unadjusted CHROMR–IFI27 association. Complete adjusted and exact fever-day-matched results are reported in **Supplementary Figure S2** and **Supplementary Table S1**. These bulk-PBMC associations do not establish cell-intrinsic regulation or causality.

**Figure 6:**
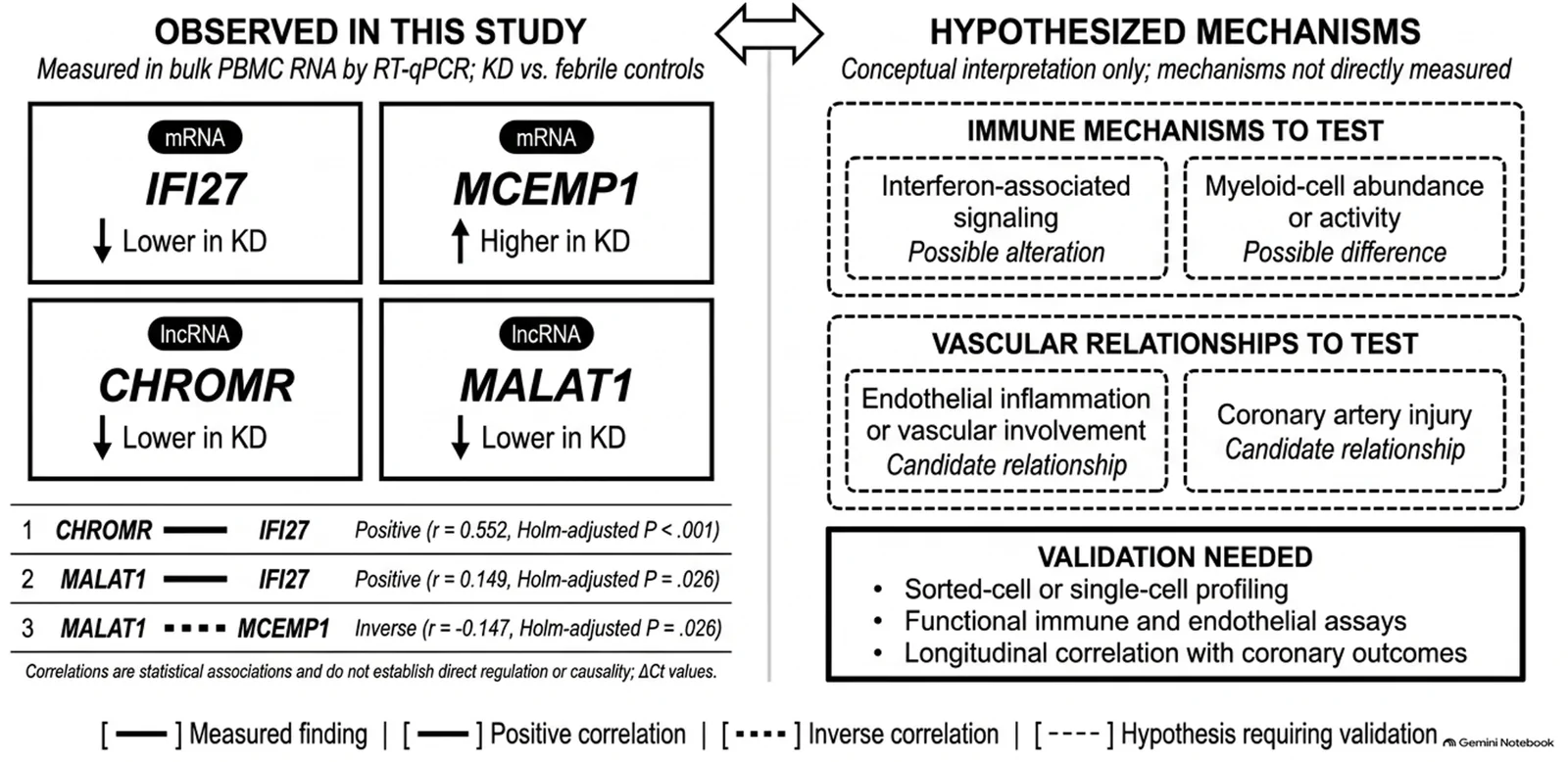
Observed bulk-PBMC transcript findings and a separate hypothesis-generating framework. A, Findings measured in this study. In unadjusted bulk-PBMC analyses, CHROMR, MALAT1, and IFI27 expression was lower and MCEMP1 expression was higher in KD than in febrile controls. After simultaneous adjustment for fever duration and broad PBMC composition, the CHROMR, IFI27, and MCEMP1 group differences remained significant, whereas the MALAT1 estimate was attenuated. Solid connectors summarize significant unadjusted transcript correlations: CHROMR–IFI27 (r=0.552; Holm-adjusted P<.001), MALAT1–IFI27 (r=0.149; P = .026), and MALAT1–MCEMP1 (r=−0.147; P = .026). Connector direction and style denote the sign of a statistical association, not causal direction or direct regulation. B, Hypotheses requiring experimental validation. Possible differences in interferon-associated signaling, myeloid-cell abundance or activity, endothelial or vascular inflammation, and coronary artery injury were not measured. Dashed boxes and connectors represent conceptual hypotheses rather than an experimentally demonstrated pathway. Validation requires cell-resolved profiling, functional assays, and longitudinal assessment of coronary outcomes. KD, Kawasaki disease; PBMC, peripheral blood mononuclear cell; RT-qPCR, reverse transcription quantitative polymerase chain reaction.

### Statistical analysis

Continuous variables were summarized as means with SDs or medians with interquartile ranges, depending on distribution. Categorical variables were summarized as counts and percentages. Between-group comparisons used t tests for approximately normally distributed continuous variables, Wilcoxon rank-sum or Mann–Whitney U tests for skewed continuous variables, and χ² or Fisher exact tests for categorical variables, as appropriate.

Diagnostic performance of the IFI27–MCEMP1 score was evaluated using receiver operating characteristic analysis. Areas under the receiver operating characteristic curve and 95% CIs were estimated using the DeLong method (13). Sensitivity, specificity, positive predictive value, and negative predictive value were calculated at the prespecified threshold of KD score ≥2.5, with exact binomial 95% CIs. Comparator-specific analyses compared KD with viral, bacterial, and other inflammatory febrile-control subgroups. Predictive values were reported only for the full febrile-control comparison because they depend on the case-control composition and may not generalize to clinical prevalence.

Differential expression of CHROMR, MALAT1, and NEAT1 between KD and febrile controls was assessed using ΔCt values and two-sided Mann–Whitney U tests. Holm adjustment controlled multiplicity across the three candidate lncRNAs. Pairwise lncRNA–mRNA associations were evaluated in the integrated same-site cohort using Pearson correlation coefficients between lncRNA ΔCt values and IFI27 or MCEMP1 ΔCt values. Holm adjustment was applied across the six lncRNA–mRNA correlations.

Associations between the KD score and candidate lncRNAs were additionally evaluated in the subset with complete paired score and lncRNA measurements (104 children; 53 with KD and 51 febrile controls). Two-sided Spearman rank correlations were calculated between the KD score and each lncRNA ΔCt value, with Benjamini–Hochberg adjustment across the three comparisons. Because higher ΔCt denotes lower expression, a positive correlation between KD score and lncRNA ΔCt indicates lower relative lncRNA expression at higher KD scores.

Univariate linear models evaluated each lncRNA or clinical variable separately and were used to estimate the proportion of KD-score variance explained. Multivariable linear regression evaluated associations between lncRNAs and the KD score after adjustment for selected clinical and laboratory covariates, including age, white blood cell count, C-reactive protein, hemoglobin, and available leukocyte differential measurements. Regression coefficients are reported with standard errors. These models assessed association with the existing score and were not interpreted as evidence of incremental diagnostic value, direct transcriptional regulation, mediation, or causality.

### PBMC-composition sensitivity analyses

Cellular-composition analyses were conducted as additional exploratory sensitivity analyses in participants with matched qPCR and complete blood count (CBC) differential data. Of 104 participants with matched data, 96 passed CBC quality-control criteria, including 50 with KD and 46 febrile controls. Records with incomplete differential components or implausible differential totals were excluded. PBMC subset-marker assays, including CD14 and CD3, and flow-cytometric subset counts were not obtained. Because RNA was measured in PBMCs, the CBC-derived monocyte fraction within the mononuclear compartment was calculated as monocytes/(monocytes + lymphocytes).

For these analyses, expression proxies were oriented so that higher values indicated greater expression and then standardized: negative Ct was used for IFI27 and MCEMP1, and negative ΔCt was used for CHROMR and MALAT1. Ordinary least-squares models estimated the standardized KD-versus-febrile-control difference for each transcript after adjustment for the standardized CBC-derived monocyte fraction. HC3 heteroscedasticity-robust standard errors were used. Additional models included standardized fever duration or calculated absolute monocyte, lymphocyte, and neutrophil counts; absolute counts were derived from the white blood cell count and corresponding differential percentages. Benjamini–Hochberg adjustment was applied across the four transcripts within each model specification. These analyses evaluated broad monocyte-versus-lymphocyte composition and were not treated as formal cell-type deconvolution. Results are shown in **Supplementary Figures S2, S3**.

### Fever-duration and matched sensitivity analyses

Fever duration at blood sampling was evaluated as a potential confounder because its distribution differed between the KD and febrile-control groups. Quality-controlled cohort sensitivity models simultaneously included diagnostic group, standardized fever duration, and the standardized CBC-derived monocyte fraction and used HC3 robust standard errors. To reduce dependence on regression extrapolation, an exact fever-day full-matching analysis was also performed within the temporal-overlap population. Exact overlap was available on fever days 3, 4, 5, 7, and 8; no quality-controlled febrile-control sample was available on fever day 6. The matched analysis included 36 participants with KD and 14 febrile controls. Weights balanced the total contribution of KD and febrile-control participants within each fever-day stratum, corresponding to an effective matched sample of 13 participants per group. Statistical significance was evaluated using 100, 000 stratified permutations, with Benjamini–Hochberg adjustment across the four transcript comparisons.

The CHROMR–IFI27 association was reassessed using partial Spearman correlation controlling for diagnostic group, fever duration, and the CBC-derived monocyte fraction in the full quality-controlled cohort. A weighted partial Spearman analysis with the same adjustment variables was performed in the exact fever-day-matched overlap population. These analyses were intended to evaluate sensitivity to sampling time and broad PBMC composition, not to establish direct or fever-independent regulation. Complete regression, matching, permutation, and partial-correlation results are reported in **Supplementary Table S1**.

### Missing data, software, and reporting standards

Analyses used complete cases without imputation; consequently, analytic sample sizes varied according to availability of the required transcript, clinical, and CBC variables. All tests were two-sided. Statistical significance was defined as P<.05 after the prespecified or analysis-specific multiplicity adjustment, where applicable. Analyses were performed using R version 4.3.

The diagnostic-validation component was reported with reference to the Standards for Reporting Diagnostic Accuracy Studies 2015 statement (14). RT-qPCR methods and assay-performance reporting were informed by the Minimum Information for Publication of Quantitative Real-Time PCR Experiments recommendations (15).

## Results

### Study population and analytic cohorts

The newly enrolled validation cohort (batch 3) included 103 children evaluated for Kawasaki disease (KD) or an alternative febrile illness: 55 with KD and 48 febrile controls. Two previously characterized same-site cohorts were included in the integrated analyses: batch 1 comprised 53 children with KD and 81 febrile controls, and batch 2 comprised 80 children with KD and 46 febrile controls. The integrated same-site cohort therefore included 188 children with KD and 175 febrile controls (363 participants) for diagnostic-score and candidate-transcript analyses (**Table 1**; **Figure 1**).

In the newly enrolled cohort, children with KD were younger than febrile controls (median age, 2.25 vs 3.79 years; P<.001) and were sampled later in the febrile illness (median, 6 vs 2 fever days; P<.001). Sex distribution was similar between groups (69.1% vs 66.7% male; P = .793). Compared with febrile controls, children with KD had lower mean hemoglobin concentrations and higher median C-reactive protein concentrations and white blood cell counts (all P<.001); platelet counts did not differ significantly (P = .070). Among the 55 children with KD, 21 (38.2%) had incomplete KD and 19 (34.5%) had coronary artery dilation or aneurysm. The 48 febrile controls included 15 (31.3%) with viral illnesses, 20 (41.7%) with bacterial illnesses, and 13 (27.1%) with other inflammatory illnesses (**Table 2**).

### Reproducibility of the prespecified IFI27–MCEMP1 score

The prespecified score, calculated as ΔCt(IFI27) − ΔCt(MCEMP1), was evaluated without refitting or threshold optimization. Higher scores represented the observed KD-associated combination of relatively lower IFI27 and higher MCEMP1 expression; they were not interpreted as a direct measure of interferon signaling or myeloid-cell activation.

In the newly enrolled cohort, the score distinguished KD from all febrile controls with an area under the receiver operating characteristic curve (AUC) of 0.88 (95% CI, 0.80–0.95). At the prespecified threshold of 2.5 or greater, sensitivity was 81.8% (95% CI, 69.1%–90.9%), specificity was 87.5% (95% CI, 74.8%–95.3%), positive predictive value was 88.2% (95% CI, 76.1%–95.6%), and negative predictive value was 80.8% (95% CI, 67.5%–90.4%) (**Table 4**; **Figure 2A**).

Comparator-specific AUCs in the newly enrolled cohort were 0.97 (95% CI, 0.93–1.00) against viral febrile controls, 0.79 (95% CI, 0.65–0.93) against bacterial febrile controls, and 0.90 (95% CI, 0.79–1.00) against other inflammatory febrile controls. In the integrated same-site cohort, the overall AUC was 0.89 (95% CI, 0.86–0.93); sensitivity was 85.6% (95% CI, 79.8%–90.3%) and specificity was 84.6% (95% CI, 78.4%–89.6%) at the same threshold. Integrated-cohort AUCs were 0.96 (95% CI, 0.93–0.98), 0.81 (95% CI, 0.73–0.88), and 0.92 (95% CI, 0.87–0.96) against the viral, bacterial, and other inflammatory comparator subgroups, respectively (**Table 4**; **Figure 2B**). Because some historical control annotations were non-mutually exclusive, the integrated subgroup results are comparator contrasts rather than prevalence estimates.

### Unadjusted candidate-lncRNA differences in the integrated cohort

In the integrated cohort, CHROMR and MALAT1 ΔCt values were higher in KD than in febrile controls, indicating lower relative expression of both transcripts in KD (Holm-adjusted P<.001 for each). NEAT1 did not differ significantly after adjustment across the three candidate lncRNAs (Holm-adjusted P = .081) (**Figure 3**; **Table 5**). These were unadjusted bulk-peripheral blood mononuclear cell (PBMC) comparisons; sensitivity analyses addressing PBMC composition and fever duration are reported below.

### Transcript associations and KD-score correlations

Across the integrated cohort (n=363), CHROMR ΔCt showed the strongest pairwise association with IFI27 ΔCt (Pearson r=0.552; Holm-adjusted P = 2.36×10^-^²^9^). Because higher ΔCt denotes lower relative expression, lower CHROMR expression tracked with lower IFI27 expression. CHROMR was not significantly associated with MCEMP1 after multiplicity adjustment (r=−0.113; Holm-adjusted P = .124). MALAT1 ΔCt was positively associated with IFI27 ΔCt (r=0.149; Holm-adjusted P = .026) and inversely associated with MCEMP1 ΔCt (r=−0.147; Holm-adjusted P = .026). NEAT1 was not associated with IFI27 (r=−0.002; Holm-adjusted P = 1.000) or MCEMP1 (r=0.056; Holm-adjusted P = .851) (**Figure 4**; **Table 5**).

In the complete paired subset of 104 children (53 with KD and 51 febrile controls), the KD score was positively correlated with CHROMR ΔCt (Spearman ρ=0.617; P = 3.19×10^-^¹²; Benjamini–Hochberg q<.001), corresponding to lower relative CHROMR expression at higher KD scores. Correlations of the score with MALAT1 ΔCt (ρ=0.191; P = .052; q=.078) and NEAT1 ΔCt (ρ=0.121; P = .222; q=.222) were not statistically significant. Complementary univariate and multivariable score-association models are summarized in **Figure 5**; these exploratory models assessed association with the existing score and not incremental diagnostic value, direct regulation, mediation, or causality.

### Sensitivity to broad PBMC composition

Of 104 participants with matched quantitative polymerase chain reaction and complete blood count differential data, 96 passed quality-control criteria (50 with KD and 46 febrile controls). The CBC-derived monocyte fraction within the mononuclear compartment differed between groups (P = .0015). After adjustment for this fraction, the standardized KD-versus-febrile-control expression differences remained significant for MALAT1 (β=−0.463 SD; 95% CI, −0.895 to −0.031; q=.042), CHROMR (β=−0.766 SD; 95% CI, −1.135 to −0.397; q=1.62×10^-4^), IFI27 (β=−1.362 SD; 95% CI, −1.674 to −1.049; q=5.77×10^-^¹³), and MCEMP1 (β=0.436 SD; 95% CI, 0.016 to 0.856; q=.042) (**Supplementary Figure S2**).

CHROMR and IFI27 group differences remained statistically robust in additional models incorporating fever duration or calculated absolute leukocyte counts, whereas the MALAT1 and MCEMP1 estimates varied more across model specifications. Marker-level relationships with the CBC-derived monocyte fraction are shown in **Supplementary Figure S3**. Because no PBMC subset markers or flow-cytometric counts were available, these analyses address only broad monocyte-versus-lymphocyte composition and cannot localize an expression difference to a specific cell population.

### Fever-duration-adjusted and exact fever-day-matched analyses

In the 96-participant quality-controlled cohort, models simultaneously adjusted for diagnosis, fever duration, and the CBC-derived monocyte fraction. The standardized KD-versus-febrile-control differences were −0.496 SD for MALAT1 (95% CI, −1.048 to 0.056; q=.077), −0.689 SD for CHROMR (95% CI, −1.100 to −0.278; q=.0025), −1.411 SD for IFI27 (95% CI, −1.758 to −1.063; q=1.10×10^-^¹¹), and 0.675 SD for MCEMP1 (95% CI, 0.243 to 1.107; q=.0034). Thus, the CHROMR, IFI27, and MCEMP1 group differences remained significant after simultaneous adjustment, whereas the MALAT1 estimate was attenuated (**Supplementary Figure S2**; **Supplementary Table S1**).

Exact fever-day overlap was available on days 3, 4, 5, 7, and 8; no quality-controlled febrile-control sample was available on day 6. The overlap population comprised 36 children with KD and 14 febrile controls, corresponding after weighting to an effective matched sample of 13 participants per group. Standardized matched differences were −0.504 SD for MALAT1 (permutation q=.100), −0.644 SD for CHROMR (q=.021), −1.332 SD for IFI27 (q=8.00×10^-5^), and 0.826 SD for MCEMP1 (q=.021). All estimates were directionally concordant with the full-cohort analysis, but MALAT1 was not significant after multiplicity adjustment (**Supplementary Table S1**).

The CHROMR–IFI27 partial Spearman correlation in the full quality-controlled cohort was 0.272 (P = .0083) after adjustment for diagnostic group, fever duration, and the CBC-derived monocyte fraction. In the exact fever-day-matched overlap population, the weighted partial correlation was attenuated and not statistically significant (ρ=0.189; P = .312). The unadjusted integrated-cohort correlation therefore did not establish a robust fever-independent or cell-composition-independent coupling between CHROMR and IFI27 (**Supplementary Table S1**).

### CHROMR assay re-evaluation and efficiency-corrected sensitivity analysis

The archived CHROMR standard curve comprised six sequential threefold dilutions measured in technical duplicate. Regression over the contiguous linear quantitative range yielded a slope of −3.406, R²=0.9982, and an amplification efficiency of 96.626% (approximately 96.6%). Technical-replicate SDs across the retained range were 0.142 to 0.391 Ct. The archived melt curve showed a single dominant peak, supporting amplification of a predominant product, but no archived gel or sequencing record was available to confirm amplicon identity (**Table 3**; **Supplementary Figure S4**).

In an assay-efficiency-corrected sensitivity analysis, lower relative CHROMR abundance in KD remained significant (P = 3.13×10^-6^), and the CHROMR–IFI27 association was essentially unchanged (Spearman ρ=−0.506 against IFI27 mean Ct; P = 4.36×10^-8^). Because a higher IFI27 Ct indicates lower IFI27 expression, the negative correlation with IFI27 Ct corresponds to a positive association between the efficiency-corrected CHROMR and IFI27 expression measures. The principal CHROMR findings therefore did not depend on assuming identical CHROMR and GAPDH amplification efficiencies.

### Measured findings and interpretive boundary

Across the unadjusted and sensitivity analyses, the most consistent findings were lower CHROMR and IFI27 expression and higher MCEMP1 expression in KD than in febrile controls. The unadjusted MALAT1 difference was less robust after fever-duration and PBMC-composition adjustment, and NEAT1 showed no significant KD-associated difference. **Figure 6A** summarizes these measured bulk-PBMC findings. The signaling, cellular, vascular, and coronary relationships in **Figure 6B** were not measured and are presented only as hypotheses requiring cell-resolved, functional, longitudinal, and independent validation.

## Discussion

In this same-site bulk-PBMC RT-qPCR study, we examined whether selected long noncoding RNAs (lncRNAs) were associated with the prespecified IFI27–MCEMP1 transcript pattern in Kawasaki disease (KD). The score reproduced discrimination between KD and febrile controls in the newly enrolled and integrated cohorts. Across the unadjusted and sensitivity analyses, the most consistent group differences were lower CHROMR and IFI27 expression and higher MCEMP1 expression in KD. MALAT1 expression was lower in the unadjusted analysis, but the estimate was attenuated after simultaneous adjustment for fever duration and broad peripheral blood mononuclear cell (PBMC) composition and was not significant after exact fever-day matching; NEAT1 showed no significant group difference. The integrated-cohort CHROMR–IFI27 correlation was strong, but it became modest after adjustment and was not significant in the restricted matched population. These findings identify an associated bulk-PBMC transcript pattern; they do not demonstrate direct lncRNA regulation, altered pathway activity, or a causal interferon–myeloid axis.

The reproducibility of the prespecified score provides a stable descriptive anchor for the exploratory lncRNA analyses. The score was applied without refitting or threshold optimization and reproduced the performance of the previously validated two-gene assay (6). IFI27 is an interferon-stimulated gene that can counter-regulate RIG-I-mediated antiviral signaling, whereas MCEMP1 has been associated with inflammatory myeloid states and is expressed in classical monocytes and macrophages in studies outside KD (7–9). Nevertheless, the abundance of one interferon-associated transcript cannot quantify global interferon activity, and MCEMP1 abundance cannot establish functional myeloid activation. The stronger score separation from viral than bacterial febrile controls may motivate host-response hypotheses, but it should not be interpreted as evidence of pathogen-specific mechanisms, particularly because historical comparator annotations were not fully mutually exclusive.

The CHROMR group difference was the most robust lncRNA finding. Lower CHROMR expression in KD remained significant after adjustment for the CBC-derived monocyte fraction, simultaneous adjustment for fever duration and PBMC composition, and exact fever-day matching. Experimental work in human macrophages has shown that CHROMR is induced by viral infection and can sequester the IRF2/IRF2BP2 transcriptional corepressor complex, thereby permitting IRF-dependent interferon-stimulated gene transcription (10). This external evidence makes the observed CHROMR–IFI27 covariation biologically plausible, but it does not establish that the same mechanism operates in KD. Indeed, the unadjusted integrated-cohort correlation was reduced after controlling for diagnostic group, fever duration, and the CBC-derived monocyte fraction, and it was not significant in the smaller fever-day-matched population. The present data therefore support a reproducible between-group CHROMR difference, but not direct CHROMR regulation of IFI27 or a robust fever-independent, cell-composition-independent coupling between these transcripts.

MALAT1 requires a more cautious interpretation. Its expression was lower in the unadjusted integrated comparison and remained significant after adjustment for broad PBMC composition alone, but the estimate was attenuated after simultaneous fever-duration and PBMC-composition adjustment and after exact fever-day matching. Its unadjusted correlations with IFI27 and MCEMP1 were statistically significant but small. In lipopolysaccharide-stimulated macrophages, MALAT1 has been reported to interact with nuclear NF-κB and modulate inflammatory cytokine expression (12). However, NF-κB activity, cytokine production, and cell-specific MALAT1 function were not measured here. MALAT1 should therefore be regarded as a secondary, model-sensitive candidate association rather than evidence of a regulatory bridge between interferon-associated and myeloid-associated biology.

NEAT1 did not differ significantly between KD and febrile controls and was not significantly associated with IFI27 or MCEMP1 after multiplicity adjustment. Experimental studies have linked NEAT1-dependent paraspeckle organization to stimulus-responsive IL8 transcription through relocation of SFPQ (11), but those findings were obtained outside KD and cannot be presumed to apply in this cohort. The absence of a NEAT1 signal indicates that the three candidate lncRNAs did not move uniformly; it does not prove KD specificity, exclude smaller NEAT1 effects, or establish that acute inflammation has no broader influence on lncRNA expression.

Cellular heterogeneity is central to interpreting every transcript result. All measurements were made in bulk PBMC RNA and may reflect both differences in cell abundance and differences in transcription within cells. Adjustment for the CBC-derived monocyte fraction suggested that broad monocyte-versus-lymphocyte composition did not fully account for the CHROMR and IFI27 group differences, whereas MALAT1 and MCEMP1 were less consistent across sensitivity models. A CBC differential cannot resolve T-cell, B-cell, natural killer-cell, or monocyte subpopulations and cannot identify the cellular source of an expression difference. In particular, higher bulk MCEMP1 expression must not be interpreted as activation per myeloid cell, even though MCEMP1 is expressed in monocytes and macrophages in other disease settings (8, 9).

Sampling time is an additional major limitation. Children with KD were sampled later in the febrile illness than febrile controls. Although the CHROMR, IFI27, and MCEMP1 group differences persisted after simultaneous regression adjustment and exact fever-day matching, temporal overlap was limited, the effective matched sample was small, and no quality-controlled febrile-control sample was available on fever day 6. Moreover, the adjusted CHROMR–IFI27 association was not confirmed in the matched population. Residual confounding by inflammatory stage, infecting pathogen, disease severity, treatment, or other time-varying immune processes therefore cannot be excluded. Prospective studies should use protocol-defined sampling windows or serial sampling across comparable fever days.

The biological framework in **Figure 6** is intentionally divided between observation and hypothesis. The measured results consist of bulk-PBMC transcript differences and statistical associations. Possible differences in interferon-associated signaling, myeloid-cell abundance or activity, endothelial or vascular inflammation, and coronary artery injury were not measured. Immune and vascular processes are central to current models of KD pathogenesis (1, 2, 4), but the present study cannot connect the measured transcripts to those downstream processes. Demonstrating such relationships would require broader interferon-stimulated gene profiling, interferon protein or downstream signaling measurements, detailed immune-cell phenotyping, endothelial or vascular assays, and longitudinal association with coronary outcomes.

The clinical role of the lncRNA findings should likewise be bounded. The IFI27–MCEMP1 score has previously been evaluated as a parsimonious diagnostic assay (6), whereas the present lncRNA analyses were exploratory and were not designed to test incremental discrimination, calibration, clinical utility, or treatment selection. The association of CHROMR with the score does not show that CHROMR improves diagnosis, and none of the candidate lncRNAs can yet define a KD endotype or predict intravenous immunoglobulin response or coronary complications. These possibilities are appropriate for future prespecified validation, but not as conclusions from the current data.

Several features strengthen the study. The diagnostic score and threshold were prespecified, limiting refitting to the newly enrolled cohort. The integrated analysis included febrile comparators representing the clinical differential diagnosis, and the candidate-transcript tests used multiplicity adjustment. The revised analysis directly evaluated two major sources of bias—broad PBMC composition and fever duration—using complementary regression and exact-matching approaches. Re-examination of the CHROMR standard curve and an assay-efficiency-corrected sensitivity analysis also showed that the principal CHROMR findings were not driven by the originally anomalous efficiency estimate. Finally, the revised interpretation explicitly separates measured findings from mechanistic hypotheses.

Important limitations remain. The observational, cross-sectional design precludes causal or temporal inference. All cohorts came from one institution, and combining same-site batches does not constitute independent external validation of the lncRNA results. The smaller quality-controlled and fever-day-overlap populations limited precision and may not represent the full cohort. Bulk-PBMC measurements, incomplete temporal overlap, and possible residual clinical confounding remain unresolved. The candidate-panel design was biologically focused rather than transcriptome-wide and could miss other regulatory RNAs. Historical febrile-control subgroup labels were not entirely mutually exclusive. The archived CHROMR melt curve and re-estimated standard curve support a predominant, quantitatively acceptable product, but gel-electrophoretic or sequence-based amplicon confirmation was unavailable; independent assay confirmation remains necessary in keeping with rigorous RT-qPCR reporting principles (15). Finally, the study did not measure pathway proteins or activity, cell-resolved expression, vascular tissue, coronary outcomes in relation to transcript levels, or incremental diagnostic utility.

Future studies should prospectively enroll multicenter KD and febrile-control cohorts with standardized fever-day sampling and independent lncRNA validation. Sorted CD14+ monocytes and defined CD3+, CD19+, and CD56+ lymphocyte populations, or single-cell RNA sequencing combined with surface phenotyping, could distinguish cellular-composition effects from cell-intrinsic transcription. Newly generated CHROMR standard curves with gel and sequence confirmation should be paired with an orthogonal expression assay. Serial sampling before and after intravenous immunoglobulin could determine whether transcript differences track inflammatory resolution, and linkage to prespecified coronary outcomes could test clinical relevance. Functional perturbation studies would then be required before proposing direct lncRNA control of IFI27, MCEMP1, or downstream vascular biology.

In conclusion, KD was associated with a bulk-PBMC transcript pattern characterized by lower CHROMR and IFI27 expression and higher MCEMP1 expression relative to febrile controls. The between-group CHROMR finding was robust across the available sensitivity analyses, whereas the CHROMR–IFI27 correlation weakened after adjustment and was not confirmed in the restricted matched population. MALAT1 was less robust, and NEAT1 showed no significant KD-associated signal. These results provide candidate lncRNA context for the IFI27–MCEMP1 transcript pattern but do not establish altered interferon signaling, functional myeloid activation, direct lncRNA regulation, or a causal pathway to coronary injury.

## Data Availability

The raw data supporting the conclusions of this article will be made available by the authors, without undue reservation.
