# Supplementary material for: Long noncoding RNA associations with an IFI27–MCEMP1 transcript pattern in Kawasaki disease
Source: Front Immunol. 2026 Sep 23;17:1882860. doi: 10.3389/fimmu.2026.1882860 (PMC13642318; doi:10.3389/fimmu.2026.1882860)

Long Noncoding RNA Associations With an IFI27–MCEMP1 Transcript Pattern in Kawasaki Disease

Supplementary Material

Short title: lncRNAs and the IFI27–MCEMP1 Pattern in KD

Table of Contents

Supplementary Methods 2
Supplementary Results 3
Supplementary Table S1. PBMC-composition, fever-duration, and exact fever-day-matched sensitivity analyses 4
Supplementary Figure S1. Representative melt curves for the MALAT1 and NEAT1 RT-qPCR assays 5
Supplementary Figure S2. Sensitivity of KD–febrile-control expression differences to PBMC-composition adjustment... 6
Supplementary Figure S3. Four-transcript expression patterns across CBC-derived PBMC composition........ 7
Supplementary Figure S4. Re-evaluation of the CHROMR RT-qPCR assay 8

Supplementary Methods

PBMC-composition and fever-duration sensitivity analyses

Cellular-composition analyses were performed in participants with matched RT-qPCR and complete blood count (CBC) differential data. Of 104 participants with matched data, 96 passed CBC quality-control criteria, including 50 children with Kawasaki disease (KD) and 46 febrile controls (FCs). Because RNA was measured in peripheral blood mononuclear cells (PBMCs), the CBC-derived monocyte fraction within the mononuclear compartment was calculated as monocytes/(monocytes + lymphocytes). Expression proxies were oriented so that higher values indicated greater expression and were then standardized: negative cycle-threshold (Ct) values were used for IFI27 and MCEMP1, and negative ΔCt values were used for CHROMR and MALAT1. Ordinary least-squares models estimated the standardized KD-versus-FC difference after adjustment for the standardized monocyte fraction, using HC3 heteroscedasticity-robust standard errors. Additional models included standardized fever duration or calculated absolute monocyte, lymphocyte, and neutrophil counts. Benjamini–Hochberg correction was applied across the four transcripts within each model specification. These analyses assessed broad monocyte-versus-lymphocyte composition and were not treated as formal cell-type deconvolution.

Fever-duration sensitivity models simultaneously included diagnostic group, standardized fever duration, and the standardized CBC-derived monocyte fraction. Exact fever-day full matching was additionally performed within the temporal-overlap population on fever days 3, 4, 5, 7, and 8; no quality-controlled FC sample was available on fever day 6. The matched analysis included 36 children with KD and 14 FCs. Weights balanced the total KD and FC contribution within each fever-day stratum, corresponding to an effective matched sample of 13 participants per group. Statistical significance was evaluated using 100,000 stratified permutations, followed by Benjamini–Hochberg correction across the four transcripts. The CHROMR–IFI27 association was reassessed by partial Spearman correlation controlling for diagnosis, fever duration, and monocyte fraction in the full quality-controlled cohort and by a weighted partial Spearman analysis with the same covariates in the matched overlap population.

CHROMR assay re-evaluation and efficiency-corrected analysis

The archived CHROMR standard curve comprised six sequential threefold cDNA dilutions measured in technical duplicate. The contiguous S2–S4 dilution levels within the linear quantitative range were retained for regression; the remaining levels are displayed for transparency. Amplification efficiency was calculated as $E (\%) = [10^{(-1/\text{slope})} - 1] \times 100$. The principal analyses used target-minus-GAPDH ΔCt values without separate gene-specific efficiency correction. A sensitivity analysis recalculated CHROMR abundance using assay-specific amplification factors derived from the CHROMR and GAPDH efficiencies. Because no archived agarose-gel image or Sanger-sequencing record was available, the CHROMR melt curve was interpreted as evidence of a predominant amplification product, not as sequence-level confirmation of amplicon identity.

Supplementary Results

PBMC-composition and fever-duration sensitivity analyses

Among the 96 participants with quality-controlled paired RT-qPCR and CBC data, adjustment for the CBC-derived monocyte fraction preserved the KD-versus-FC direction for all four transcripts (Supplementary Figures S2 and S3). The standardized KD coefficients (95% CI; Benjamini–Hochberg q value) were −0.463 (−0.895 to −0.031; $q = 0.042$) for MALAT1, −0.766 (−1.135 to −0.397; $q = 1.62 \times 10^{-4}$) for CHROMR, −1.362 (−1.674 to −1.049; $q = 5.77 \times 10^{-13}$) for IFI27, and 0.436 (0.016 to 0.856; $q = 0.042$) for MCEMP1. Thus, the observed KD-associated pattern was not explained solely by the broad monocyte-versus-lymphocyte composition captured by the CBC differential.

After additional adjustment for fever duration, the directions remained unchanged: MALAT1 $\beta = -0.496$ (95% CI, −1.048 to 0.056; $q = 0.077$), CHROMR $\beta = -0.689$ (95% CI, −1.100 to −0.278; $q = 0.0025$), IFI27 $\beta = -1.411$ (95% CI, −1.758 to −1.063; $q = 1.10 \times 10^{-11}$), and MCEMP1 $\beta = 0.675$ (95% CI, 0.243 to 1.107; $q = 0.0034$) (Supplementary Table S1, panel A). Exact fever-day matching likewise preserved the direction of all four differences. Permutation-based q values were 0.100 for MALAT1, 0.021 for CHROMR, 8.00×10^{-5} for IFI27, and 0.021 for MCEMP1.

In the full quality-controlled cohort, the CHROMR–IFI27 association remained detectable after adjustment for diagnosis, fever duration, and monocyte fraction (partial Spearman $\rho = 0.272$; $P = 0.0083$). In the smaller exact fever-day-matched overlap population, the estimate remained positive but was less precise and was not statistically significant (weighted partial Spearman $\rho = 0.189$; $P = 0.312$) (Supplementary Table S1, panel B). These sensitivity analyses support robustness of the direction of association but do not establish direct regulation.

CHROMR assay re-evaluation and efficiency-corrected analysis

Reanalysis of the archived CHROMR standard-curve data identified the contiguous S2–S4 linear range, with a fitted slope of −3.406, $R^2 = 0.9982$, and an amplification efficiency of 96.626% (approximately 96.6%). Technical-duplicate SDs across the retained range were 0.142 to 0.391 Ct (Supplementary Figure S4). GAPDH amplification efficiency was 94.041%. When CHROMR abundance was recalculated using assay-specific amplification factors, lower CHROMR expression in KD remained significant ($P = 3.13 \times 10^{-6}$), and its association with IFI27 mean Ct was essentially unchanged (Spearman $\rho = -0.506$; $P = 4.36 \times 10^{-8}$). Because a higher IFI27 Ct indicates lower IFI27 expression, this negative Ct-scale correlation corresponds to a positive expression-scale association. The primary CHROMR conclusions therefore did not depend on assuming identical CHROMR and GAPDH amplification efficiencies.

Supplementary Table S1. PBMC-composition, fever-duration, and exact fever-day-matched sensitivity analyses

Panel A. Transcript-level sensitivity analyses

Transcript	PBMC composition + fever adjusted β (95% CI)	BH q	Exact fever-day matched β	Permutation P	BH q
MALAT1	−0.496 (−1.048 to 0.056)	0.077	−0.504	0.100	0.100
CHROMR	−0.689 (−1.100 to −0.278)	0.0025	−0.644	0.0129	0.021
IFI27	−1.411 (−1.758 to −1.063)	1.10×10^{-11}	−1.332	2.00×10^{-5}	8.00×10^{-5}
MCEMP1	0.675 (0.243 to 1.107)	0.0034	0.826	0.0158	0.021

Panel B. CHROMR–IFI27 partial-correlation sensitivity analyses

CHROMR–IFI27 analysis	N/analysis population	Partial Spearman ρ	P
Full cohort, adjusted for diagnosis, fever duration, and PBMC monocyte fraction	96	0.272	0.0083
Exact fever-day-matched overlap population; weighted analysis with the same covariates	36 KD/14 FC; effective 13/group	0.189	0.312

Table notes

β values are standardized KD-versus-FC expression differences; negative values indicate lower relative expression in KD. Full-cohort models used HC3 heteroscedasticity-robust standard errors and included the CBC-derived monocyte fraction and fever duration. Exact fever-day full matching used fever days 3, 4, 5, 7, and 8 and 100,000 stratified permutations. Benjamini–Hochberg correction was applied across the four transcripts. In panel B, the full-cohort partial Spearman analysis adjusted for diagnosis, fever duration, and monocyte fraction; the matched analysis used the same covariates with matching weights. CBC, complete blood count; CI, confidence interval; FC, febrile control; KD, Kawasaki disease; PBMC, peripheral blood mononuclear cell.

Supplementary Figure S1. Representative melt curves for the MALAT1 and NEAT1 RT-qPCR assays.

Post-amplification melt-curve analysis showed a single dominant melting peak for MALAT1 (A) and NEAT1 (B), supporting amplification of one predominant product under the assay conditions. Melt-curve analysis does not provide sequence-level confirmation of amplicon identity. RT-qPCR, reverse transcription quantitative polymerase chain reaction.

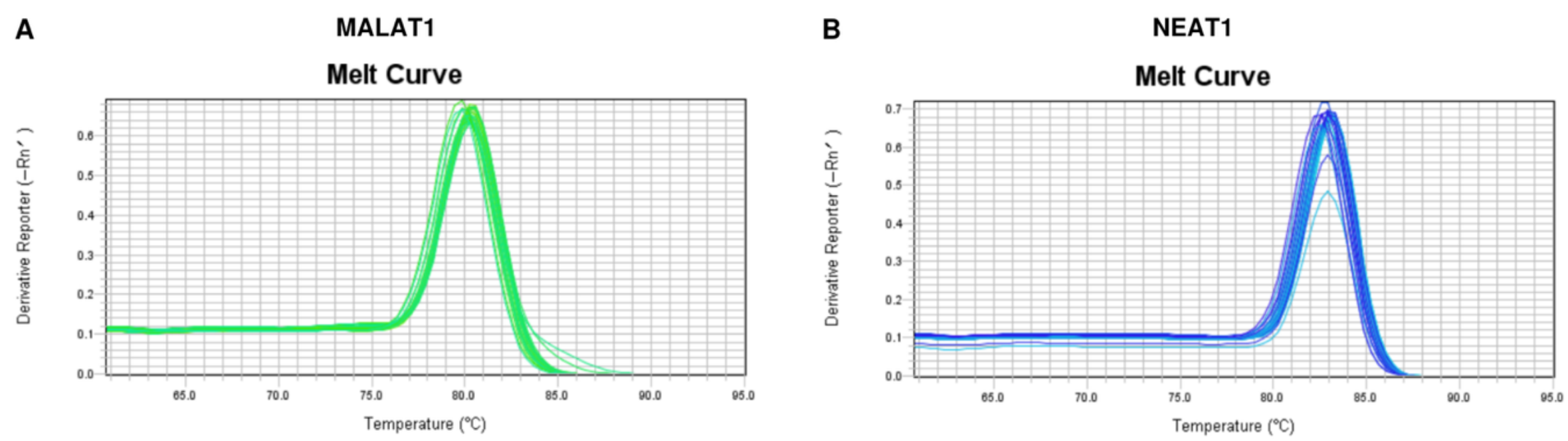

Supplementary Figure S2. Sensitivity of KD–febrile-control expression differences to PBMC-composition adjustment.

Standardized expression proxies were defined as negative Ct for IFI27 and MCEMP1 and negative ΔCt for MALAT1 and CHROMR, so that higher values indicate greater relative expression. Points show standardized KD-versus-febrile-control coefficients and bars show 95% CIs for the unadjusted model, the model adjusted for the CBC-derived monocyte fraction within the mononuclear compartment, the model additionally adjusted for fever duration, and the model adjusted for calculated absolute leukocyte counts. Negative coefficients indicate lower relative expression in KD. Benjamini–Hochberg correction was applied across the four transcripts within each model specification. Exact fever-duration-adjusted estimates are reported in Supplementary Table S1. CBC, complete blood count; CI, confidence interval; Ct, cycle threshold; KD, Kawasaki disease; PBMC, peripheral blood mononuclear cell.

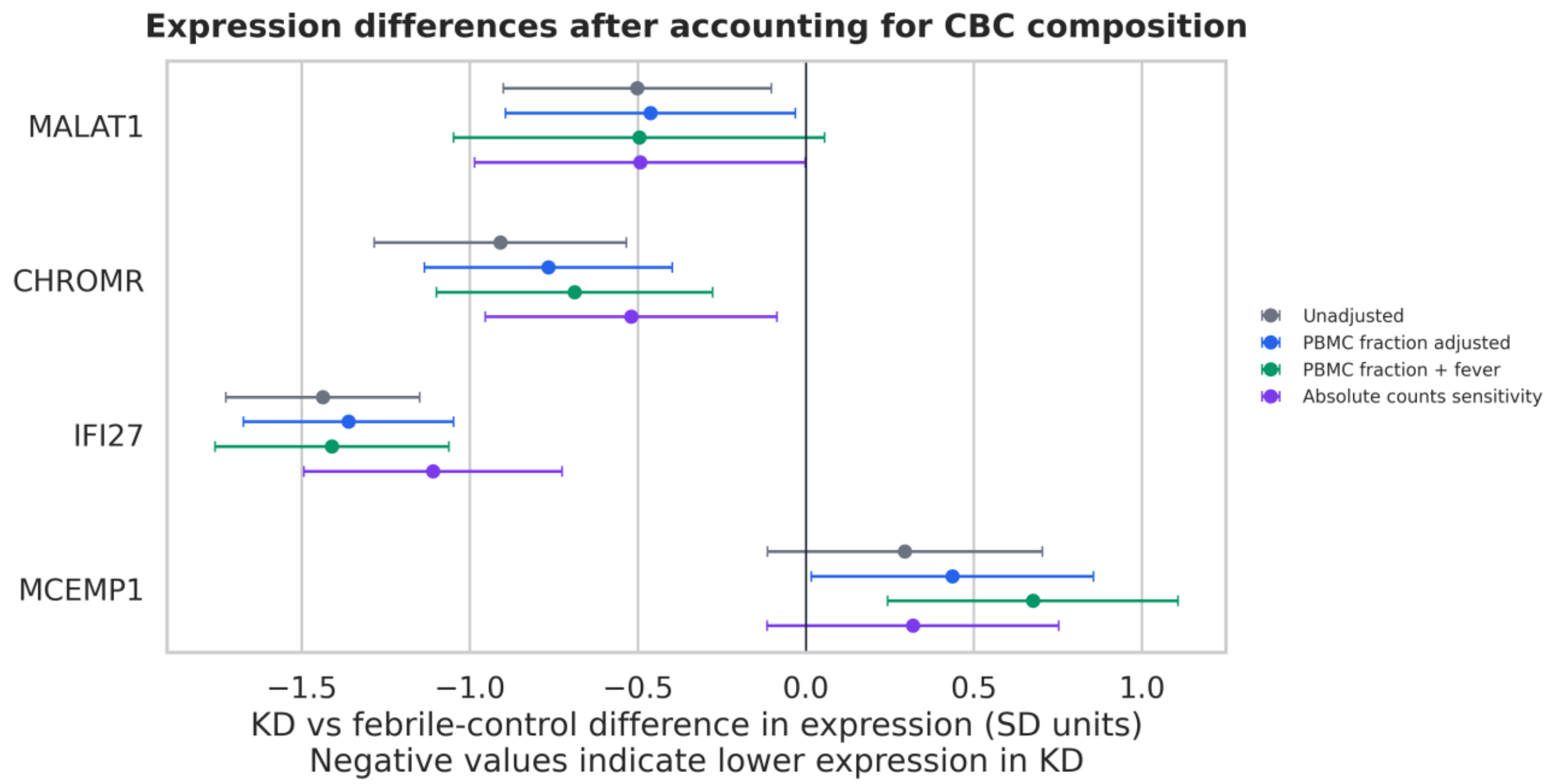

Supplementary Figure S3. Four-transcript expression patterns across CBC-derived PBMC composition.

Standardized expression proxies are plotted against the monocyte fraction among mononuclear cells, with separate descriptive regression lines for Kawasaki disease and febrile controls. Each panel displays the standardized KD coefficient and nominal two-sided P value from the ordinary least-squares model adjusted for the CBC-derived monocyte fraction; multiplicity-adjusted q values are reported in the Supplementary Results. These CBC-based analyses capture broad monocyte-versus-lymphocyte composition and are not formal cell-type deconvolution. CBC, complete blood count; KD, Kawasaki disease; PBMC, peripheral blood mononuclear cell.

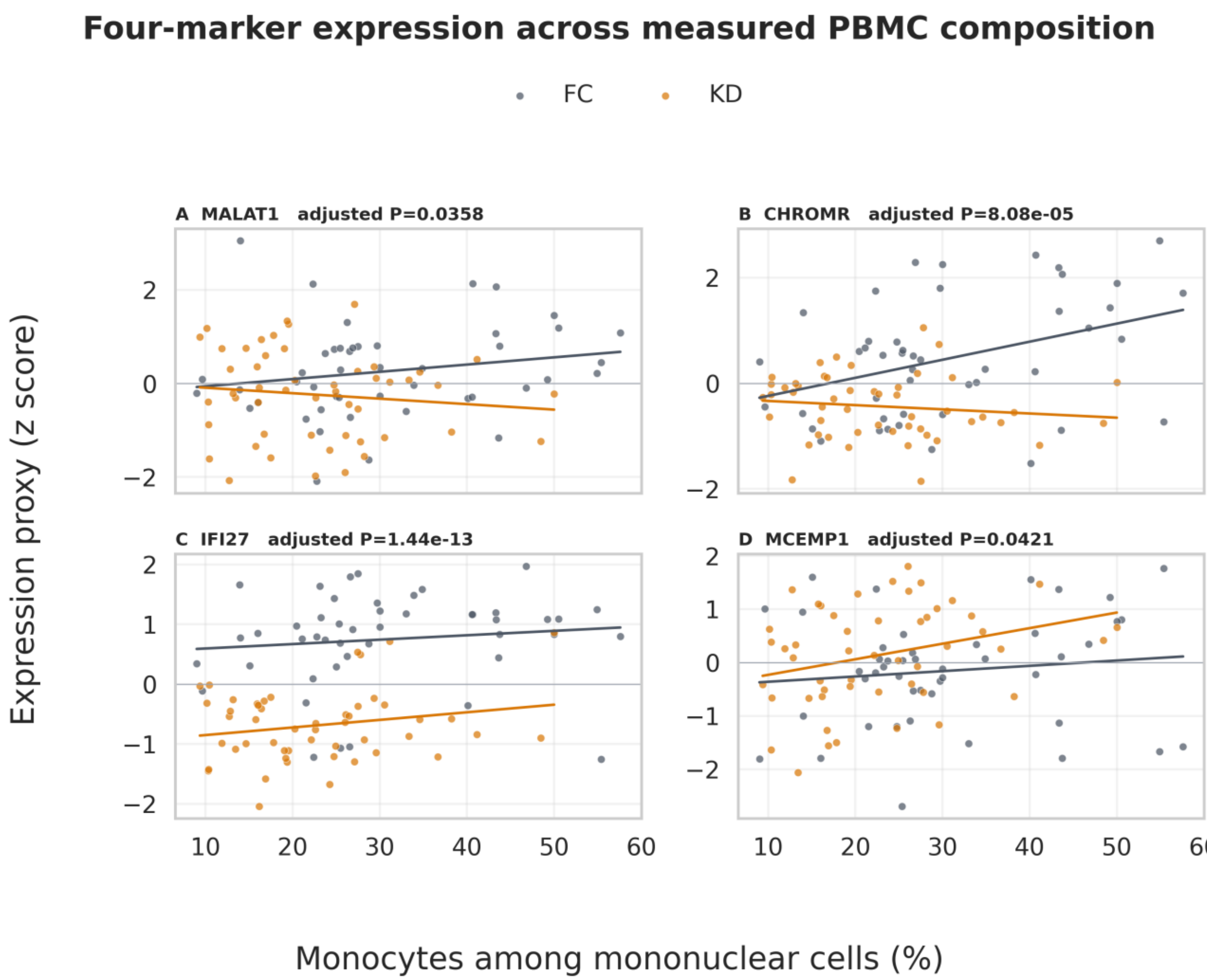

Supplementary Figure S4. Re-evaluation of the CHROMR RT-qPCR assay.

(A) Standard-curve data from six sequential threefold cDNA dilutions measured in technical duplicate. Mean Ct values ± SD are shown. Regression over the retained contiguous S2–S4 linear range yielded a slope of −3.406, $R^2 = 0.9982$, and an amplification efficiency of 96.626%, calculated as E (%) = [10^(−1/slope) − 1] × 100. Remaining dilution levels are shown for transparency but were not included in the reported regression. (B) Technical-duplicate SD at each dilution level; SDs across the retained S2–S4 range were 0.142 to 0.391 Ct. (C) Representative archived CHROMR melt curve showing one dominant peak, supporting amplification of a predominant product. No archived gel-electrophoresis image or sequencing record was available; therefore, panel C should not be interpreted as sequence-level confirmation of amplicon identity. Ct, cycle threshold; RT-qPCR, reverse transcription quantitative polymerase chain reaction.

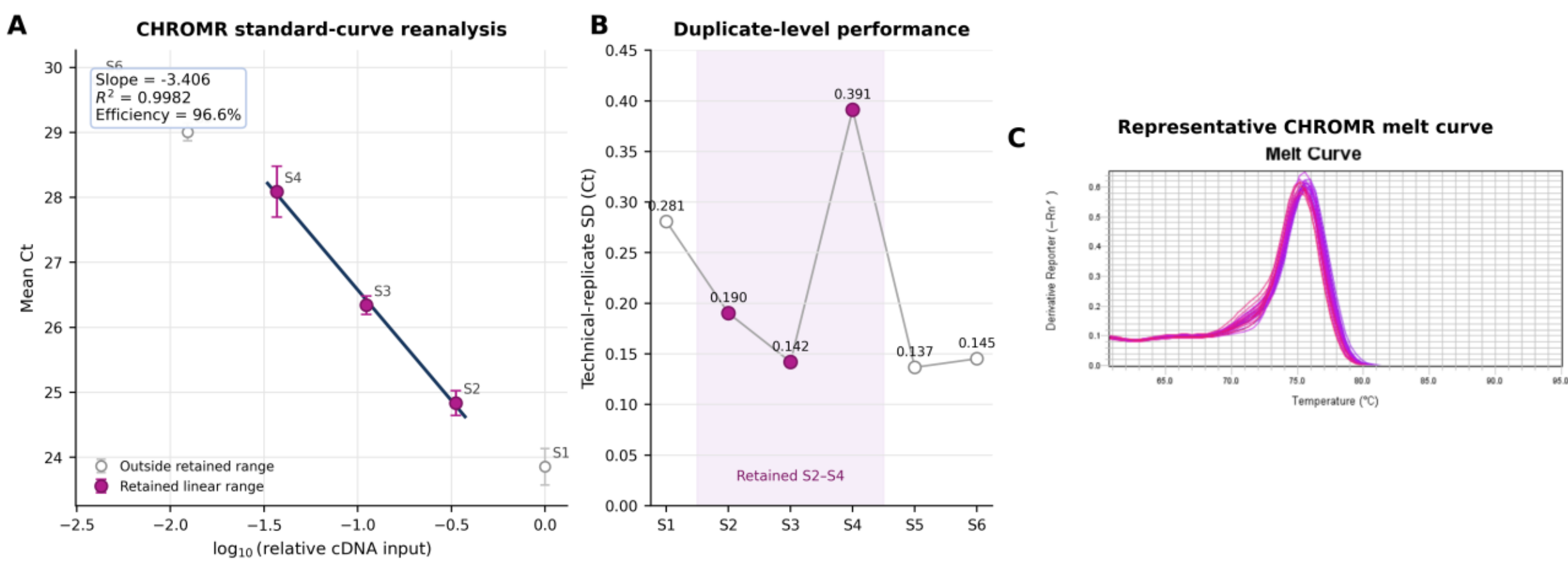

Supplement: Supplementary file 1 [file Presentation1.pdf]
